# A 29-Year-Old With Iron Deficiency and Multifocal Cerebral Infarcts

**DOI:** 10.1016/j.chpulm.2023.100021

**Published:** 2023-09-14

**Authors:** Erika Becerra-Ashby, Tiffany Gardner, Kelli Robertson, Katie E. Raffel, Peter Hountras

**Affiliations:** a Departments of Medicine-Pediatrics, University of Colorado, Denver, CO; b Medicine, University of Colorado, Denver, CO; c The Institute for Healthcare Quality Safety and Efficiency, University of Colorado, Denver, CO; d Departments of Pulmonary & Critical Care Medicine, University of Colorado, Denver, CO; e Pulmonary Vascular Disease, University of Colorado, Denver, CO

## Abstract

A 29-year-old man with a history of mood disorder was admitted with acute encephalopathy after friends had requested a welfare check. He initially was disoriented, with poor recall and reporting delusions of alien interaction. During interview, he was falling asleep intermittently but was able to be aroused. He denied any new symptoms except discomfort with eating because of insects inside his body. His friend reported the patient used alcohol 1 to 2 drinks weekly, and LSD and marijuana use the weekend prior. He also has a remote history of recreational cocaine use in college that stopped because of nosebleeds. The patient reported a daily medication for anxiety, but he was unable to recall the name.

## Physical Examination Findings

The patient was found by emergency medical services to be hypoxic to 85% on room air requiring 1 L of oxygen and tachycardic to 130 beats/min. On arrival to the ED, his saturations improved to 91% on room air, tachycardia decreased to 107 beats/min, and he had mild tachypnea with a respiratory rate of 23 breaths/min and BP of 95/61 mm Hg. On examination, the patient appeared anxious but not in acute distress or ill appearing. Cardiovascular and pulmonary examination showed cleared lung fields bilaterally and regular rate/rhythm without murmurs or lower extremity edema. No skin lesions or rashes were noted. His head was normocephalic, with clear oropharynx, moist mucus membranes, and clear conjunctivae. On neurologic examination, he was alert and oriented to self and location having tangential thoughts. Reflexes and cranial nerves were intact without deficit, dysarthria, or facial asymmetry. No sensory deficit or motor weakness was appreciated. An ataxic gait was noted.

## Diagnostic Studies

Laboratory results on admission were remarkable for urine toxicology positive for tetrahydrocannabinol and severe iron deficiency anemia with ferritin undetectable. His comprehensive metabolic panel and thyroid stimulated hormone were within normal limits. Chest radiograph showed an incidental mass on the dome of the liver suspected to be calcification, but it was otherwise normal. CT scan of the head did not demonstrate any acute abnormalities. A lumbar puncture was performed with no abnormal findings. Psychiatry was consulted in the ED and shared concern for an atypical presentation of schizophrenia vs substance-induced psychosis. They recommended further history per family to assist in diagnosis and initiation of risperidone. Patient was admitted awaiting inpatient psychiatric bed availability.

**Figure 1:**
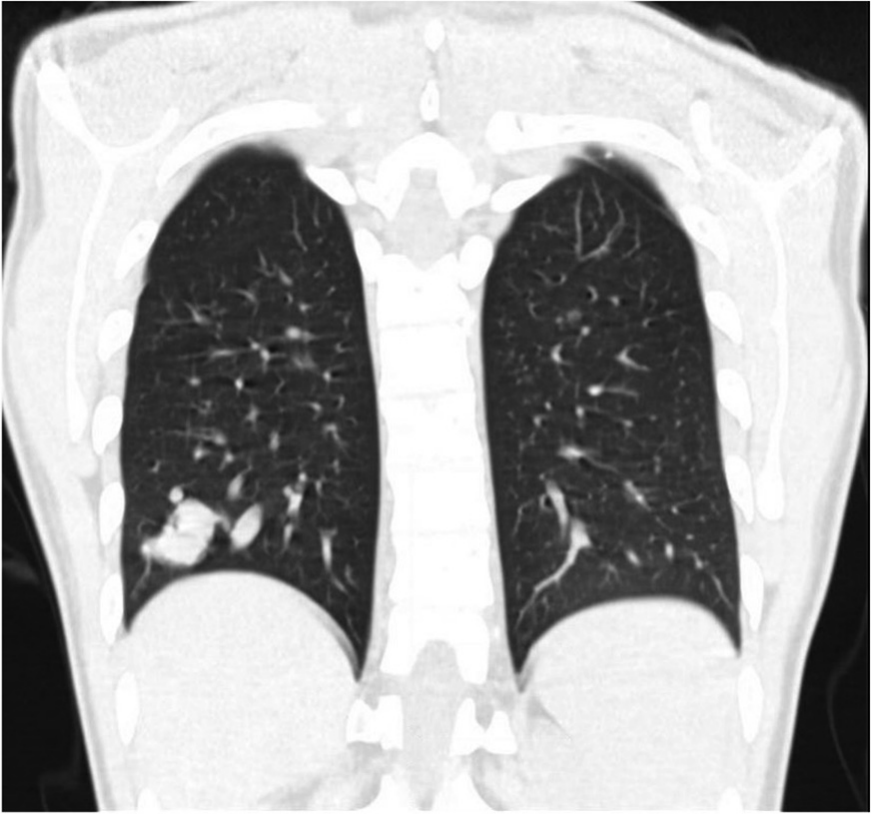
CT chest scan, coronal view: right lower lobe pulmonary arteriovenous malformation measuring 2.6 cm.

**Figure 2:**
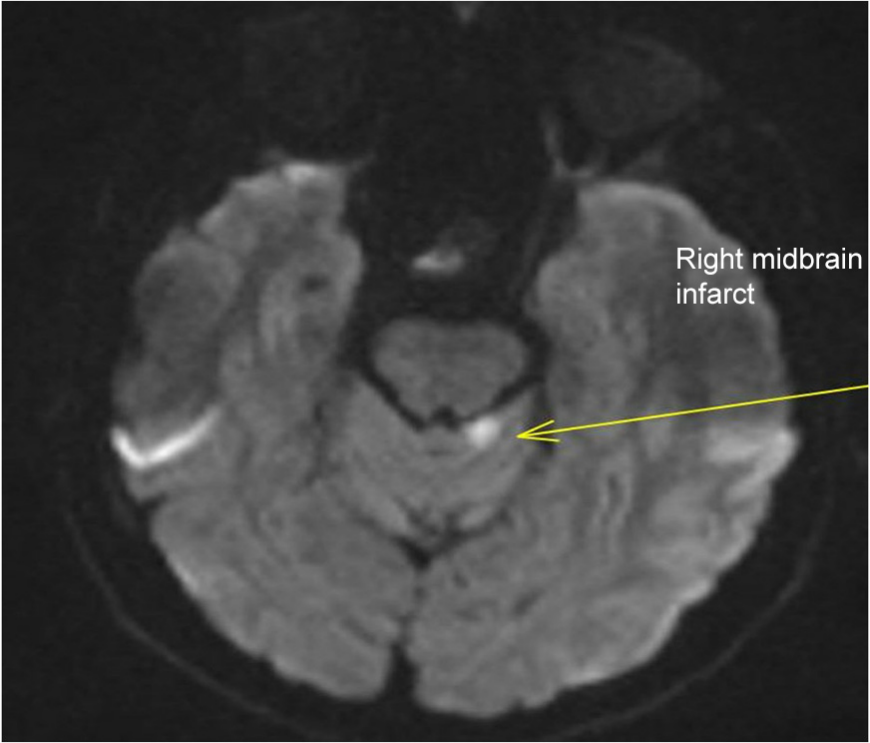
MRI brain noncontrast scan, axial T1 view: multifocal infarcts of the left superior cerebellum, right midbrain, and bilateral ventral medial thalami.

After 3 days of antipsychotic treatment with escalating dosing, the patient remained without improvement to mentation. Given lack of improvement in conjunction with severe iron deficiency anemia without clear cause, further investigation of the mass on the dome of the liver was performed. While awaiting further workup, the mother of the patient was reached and reported a history of hereditary hemorrhagic telangiectasia (HHT) in herself. A CT scan of the chest/abdomen/pelvis was obtained, demonstrating a large right lower pulmonary arteriovenous malformation measuring approximately 2.6 cm and a small renal and liver infarct (Fig 1). Subsequent urgent MRI scan of the brain was obtained showing late acute multifocal infarcts of the left superior cerebellum, right midbrain, and bilateral ventral medial thalami (Fig 2).

*What is the diagnosis?*

*Diagnosis:* Multifocal brain infarcts due to pulmonary arteriovenous malformation in the setting of HHT

## Discussion

Pulmonary arteriovenous malformations (PAVMs) are abnormal communications between pulmonary arteries and veins. PAVMs are rare in the general population. They can be caused by acquired medical conditions, such as hepatic cirrhosis, and complications of surgical interventions, including congenital heart surgeries. Less commonly, PAVMs can be associated with penetrating chest trauma, mitral stenosis, schistosomiasis, actinomycosis, Fanconi syndrome, and metastatic thyroid carcinoma. However, the vast majority of PAVMs are congenital due to HHT. HHT accounts for approximately 70% of PAVM cases, with some HHT centers reporting prevalence as high as 90% to 95% given the underreported nature of the disease. PAVMs can cause life-threatening complications (eg, stroke, brain abscess). The risk of these complications is substantial, with up to 40% of patients with PAVMs developing embolic stroke or brain abscess.

HHT is an autosomal dominant disease. It is a clinical diagnosis by meeting three of four Curaçao criteria. The criteria are as follows: (1) spontaneous and recurrent epistaxis (as measured by the epistaxis severity score), (2) multiple mucocutaneous telangiectasias at characteristic sites (tongue, buccal mucosa, and fingertips), (3) visceral lesions at characteristic sites (GI, pulmonary, cerebral, or spinal arteriovenous malformations [AVMs]), and (4) first-degree relative with HHT. Most cases of HHT are caused by mutations in the *ENG* gene and activin type-II-like receptor kinase 1, causing HHT type 1 and HHT type 2, respectively. If the Curaçao criteria are not met, which can be the case in mild presentations because of incomplete penetrance, genetic testing can be performed to confirm diagnosis. Genetic testing can also give us insight into the complications that can manifest, with HHT type 1 being more associated with cerebral and pulmonary AVMs than HHT type 2, which is more associated with GI/liver AVMs and pulmonary hypertension. Given this varying degree of clinical manifestation, it is important to begin surveillance at a young age. For this reason, genetic testing is offered to all patients with HHT and their children. The high occurrence of PAVMs in HHT coupled with the high morbidity and mortality they carry highlight the importance of routine screening starting at the time of diagnosis. After a negative test, it is recommended that a PAVM screen is repeated every 3 to 5 years.

Treatment of PAVMs is dependent on size of the feeding artery. For patients with feeding artery ≤ 2 mm, surveillance is every 3 to 5 years with CT angiography of the chest because pulmonary AVM growth is very slow. If feeding artery is ≥ 2 mm, embolization is the current treatment of choice. For acute hemorrhage or repeat failed embolization (ie, two or more), surgical excision of PAVM is the alternative treatment; however, this is rare. After interventional therapy, patients remain at risk of air embolism and cerebral abscess given their continued presence of shunting on echocardiogram. For this reason, it is recommended patients refrain from scuba diving, 0.22-μm filters are placed on all IV drips, and patients are treated with antibiotics prophylactically prior to any oral procedure, including dental procedures.

### Clinical Course

The patient underwent echocardiogram with bubble and chest CT angiography demonstrating a ≥ 2-mm feeding artery and the presence of right-left shunting. He underwent embolization of his PAVM with interventional radiology. His procedure was performed without complications.

After discharge, the patient was referred to a regional HHT center of excellence. He was screened for HHT based on Curaçao criteria. He was found to meet three of four criteria including epistaxis (epistaxis severity score > 2), pulmonary AVM, and first-degree relative. Given this diagnosis, it was determined that this patient’s PAVM was caused by HHT. His oral iron was switched to IV iron, and risperidone was discontinued. Over the following 4 months, the patient’s mentation improved, which was attributed to poststroke psychosis, allowing him to return to his independent living and career as a software engineer.

## Clinical Pearls

1. *PAVMs are a rare communication between pulmonary arteries. Although rare in the general population, they are frequently present in HHT. Any patient diagnosed with PAVM should be screened and evaluated for HHT.*
2. *HHT is an autosomal dominant disease. Diagnosis is based on meeting at least three of four Curaçao criteria. Genetic testing can be confirmatory and can assist with management. Patients diagnosed with HHT should be referred to a regional HHT center of excellence.*
3. *PAVMs carry a high risk of stroke and brain abscess given their shunt physiology. A PAVM screen through echocardiogram with bubble is recommended at the time of diagnosis in all patients with HHT.*
4. *Treatment of PAVMs includes embolization for all PAVMs with a feeding artery ≥ 2 mm.* *Bevacizumab, a vascular endothelial growth factor inhibitor, has emerged as a potential symptomatic treatment for HHT, particularly in cases of high cardiac output heart failure and epistaxis. Therefore, it may have a role in PAVMs; however, more research needs to be done. Lifestyle modifications are also recommended, which include refraining from scuba diving, using 0.22-μm filters on all IV drips, and using prophylactic antibiotics prior to any oral procedure, including dental procedures.*

## Financial/Nonfinancial Disclosures

None declared.
